# Psychiatric liaison referrals and the 4PM Rush

**DOI:** 10.1192/bjo.2021.956

**Published:** 2021-06-18

**Authors:** Kaj Svedberg

**Affiliations:** 1 Rawalpindi Medical University

## Abstract

**Aims:**

Referrals to the psychiatric Liaison team in A&E seem to come in the afternoon in kismet as the day shift is ending. This study looked at the timing distribution of referrals to try improve amount of jobs being handed over to the evening shifts.

**Method:**

Referrals made to Homerton University Hospital (HUH) psychiatric liaison was parsed into 1 hour bins and plotted as a histogram (data between August 2016–October 2019. N = 14182). The data were compared to diurnal human body temperature variation, as well as data published on Hospital Accident & Emergency Activity 2019–20 (digital.nhs.uk) for Ambulance attendances.

**Result:**

Referrals to HUH liaison team appear to closely follow the average human body temperature variations per hour (Pearson Correlation coefficient = 0.90). A peak appears to occur around 4 PM, and a low at 7AM. The referrals data also mirrored timings of official Hospital Episode Statistics (HES) reports 2019–2020 for ambulance attendance in England (Pearson Correlation coefficient = 0.94).

**Conclusion:**

Attendance to A&E and referrals to psychiatric liaison appear correlated to a circadian bound rhythm. “The 4PM referrals rush” appears to be a genuine phenomenon replicated in not only HUH mental health referrals, but general ambulance attendance throughout all of England. The body temperature analogue for circadian rhythm may be humorously applied here to correlate with the increased referral rates to A&E; the emergency department could be said to be truly heating up in the afternoon. Indeed temperature and activity has already been shown to link strongly via the Arrhenius equation in cricket activity such as chirps per minute. The conclusions drawn here are that acute mental health attendances, like general health attendances as a whole follow underlying but powerful patterns, and provisions might best be allocated to address this rather than thinking of fixed 9-5 working schedules.

